# Signaling pathways have an inherent need for noise to acquire information

**DOI:** 10.1186/s12859-020-03778-x

**Published:** 2020-10-16

**Authors:** Eugenio Azpeitia, Eugenio P. Balanzario, Andreas Wagner

**Affiliations:** 1 Department of Evolutionary Biology and Environmental Studies, University of Zürich, Zurich, Switzerland; 2 Swiss Institute of Bioinformatics, Lausanne, Switzerland; 3 Centro de Ciencias Matemáticas, Universidad Nacional Autónoma de México, Morelia, Mexico; 4 The Santa Fe Institute, Santa Fe, NM USA

**Keywords:** Information acquisition in living organisms, Signaling pathways, Noise and information, Reversible binding reactions, Stochastic processes

## Abstract

**Background:**

All living systems acquire information about their environment. At the cellular level, they do so through signaling pathways. Such pathways rely on reversible binding interactions between molecules that detect and transmit the presence of an extracellular cue or signal to the cell’s interior. These interactions are inherently stochastic and thus noisy. On the one hand, noise can cause a signaling pathway to produce the same response for different stimuli, which reduces the amount of information a pathway acquires. On the other hand, in processes such as stochastic resonance, noise can improve the detection of weak stimuli and thus the acquisition of information. It is not clear whether the kinetic parameters that determine a pathway’s operation cause noise to reduce or increase the acquisition of information.

**Results:**

We analyze how the kinetic properties of the reversible binding interactions used by signaling pathways affect the relationship between noise, the response to a signal, and information acquisition. Our results show that, under a wide range of biologically sensible parameter values, a noisy dynamic of reversible binding interactions is necessary to produce distinct responses to different stimuli. As a consequence, noise is indispensable for the acquisition of information in signaling pathways.

**Conclusions:**

Our observations go beyond previous work by showing that noise plays a positive role in signaling pathways, demonstrating that noise is essential when such pathways acquire information.

## Background

Information about the environment is fundamental when living organisms make decisions that affect their survival and reproduction [1]. For example, microbes detect nutrients and respond by adjusting their growth rate, animals detect predators and respond by fleeing, and plants detect herbivores and respond by synthesizing defense chemicals.

At the cellular level, signaling pathways are the main molecular mechanism by which organisms acquire information. They typically detect the presence of a molecular signal or cue [2] about the environment through the binding of this molecule to a receptor. Once the signal has been detected, a chain of intermediary events transmits this information to the cell’s interior, where it ultimately regulates gene expression. Signaling pathways vary widely, including in their number of molecular interactions, signal and receptor affinities, the presence of feedback and feed-forward interactions, and the number of regulated genes [3, 4]. However, they all share some elementary processes, such as the reversible binding of molecules, which is necessary to detect a signal by a receptor, transmit its presence via effector molecules, for example through allosteric control of these molecules, and regulate gene expression through the DNA binding of molecules such as transcription factors and RNA polymerases.

Noise is present at all spatial and temporal scales of biological organization, from population dynamics to molecular interactions, including signaling pathways [5, 6]. Its main source is fluctuations, either in the environment or in the internal state of an organism or cell, including fluctuations in temperature, nutrient availability, or in the concentration, movement, activity, and interactions of molecules [6–12].

Noise can interfere with organisms by hampering their ability to acquire information necessary for responding optimally to their environment. However, noise can also enhance the acquisition of information [13–17]. For example, it can improve the detection of weak signals, a phenomenon known as subthreshold stochastic resonance [18, 19]. Noise can also improve the detection of strong signals (suprathreshold stochastic resonance) through redundant pathways for signal detection [9, 20–22]. Previous work has shown that non-minimal levels of noise are observed at optimal rates of information transmission, and that noise can increase the information capacity of cells [10, 23–25]. Even in cases where noise is detrimental for the acquisition of information at the individual level, it can improve the detection of a signal at the population level [26].

**Fig. 1:**
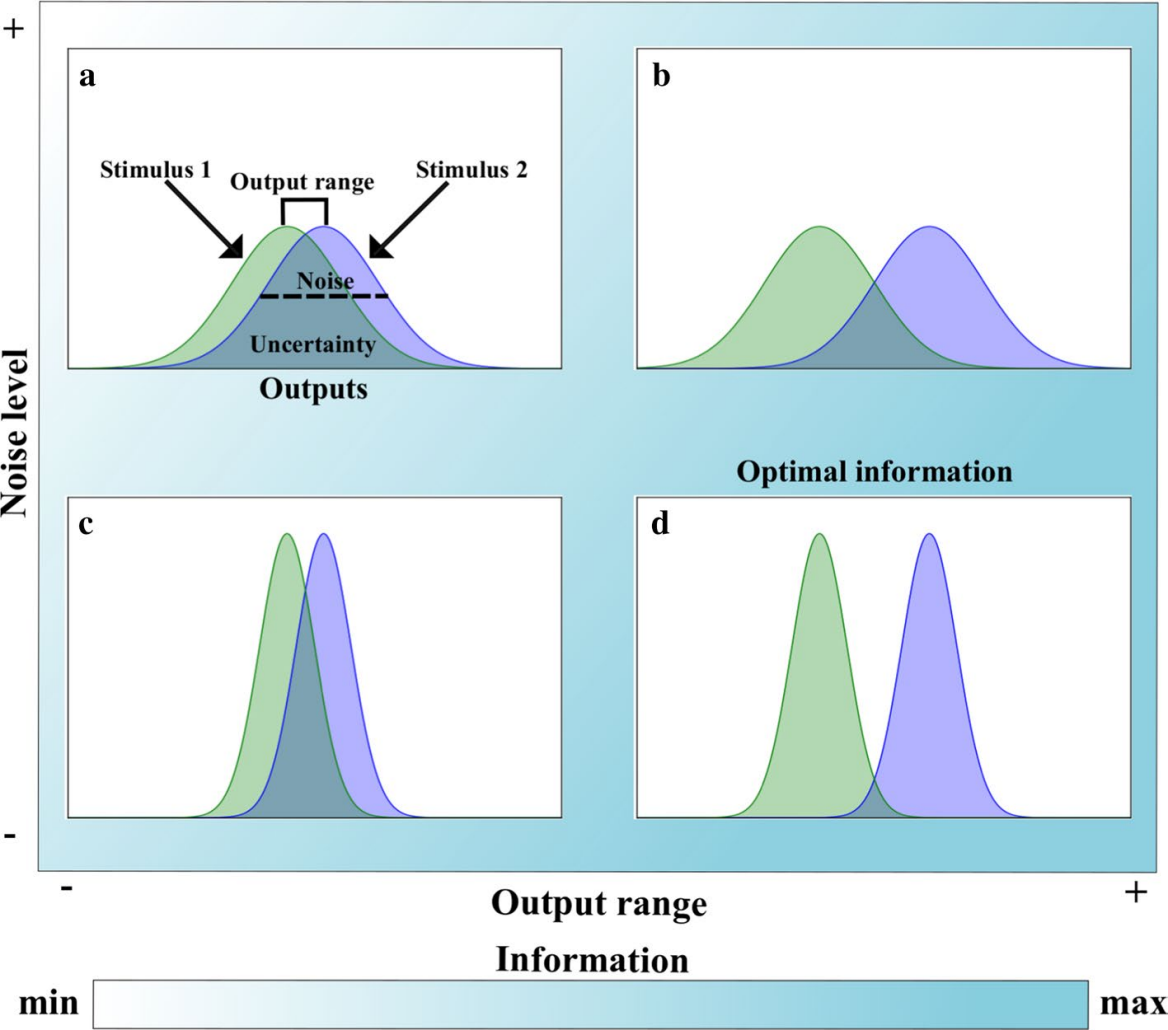
Relationship of acquired information with both noise and output range. Example of two hypothetical stimuli that produce different but overlapping response distributions (green and blue distributions). The amount of information acquired at different levels of noise (*y* axis) and with different output ranges (*x* axis) is indicated by the color bar. The black dashed line in **a** is a schematic representation of noise (i.e., the standard deviation of the response distributions, green and blue). The square bracket above the response distributions in **a** indicates the output range (i.e., the maximal difference of the mean values of the response distributions). Increasing the output range **b** and reducing noise **c** decrease the overlap (uncertainty) between response distributions observed in **a**. **d** Acquired information is largest (maximal) when noise is minimized and the output range is maximized

These observations stand in contrast to predictions of classical information theory, which posit that noise degrades the capability of a communication channel to transmit information. For example, in simple systems such as a binary or a symmetric information transmission channel, the maximum capacity of the channel to transmit information can only be realized in the absence of noise [27]. In signaling pathways, noise transforms a stimulus, such as the concentration of a nutrient, into a distribution of outputs or responses. Any overlap between the response distributions produced by two different stimuli, such as two different signal concentrations, creates uncertainty about which stimuli produced which output [28] (Fig. 1a). Noise increases the overlap between response distributions, unless the response distributions can be made more distinct by separating their means while preserving their dispersion (e.g., their variance) [11]. This implies that to acquire more information, either the range of outputs produced by different stimuli must increase (Fig. 1b), or noise must decreases (Fig. 1c). In this scenario information acquisition is maximized when the output range is maximized and noise is minimized (Fig. 1d).

Because information acquisition is important for evolutionary adaptation [29–31], natural selection must have tuned the kinetic parameters of molecular interaction in signaling pathways to either increase the benefits of noise, or to reduce its negative effects. While a few studies have explored the effect of kinetic parameters on information transmission in signaling pathways, small gene networks, and gene expression systems [10, 11, 24, 32], most of these studies did not explicitly model the molecular interactions involved in signaling. Therefore, they provide little intuition about why and how the kinetic properties of molecular processes affect information acquisition.

Here we model the kinetic properties of reversible binding interactions, which are central for all signaling pathways, to study the relationship between noise, output range and information acquisition. To do this, we first study the relationship between these quantities in the reversible binding of two molecules that represent a signal and a receptor. We then analyze how information is transmitted in a chain of consecutive binding interactions. Subsequently, we focus on information acquisition in gene regulation by the reversible binding of a transcription factor (TF) to DNA. Finally, we analyze the effect of extrinsic noise. Our results show that under a broad range of biochemically sensible parameters and biological conditions, a noisy dynamic is essential to produce an output whose range depends on the intensity of different stimuli, which is necessary for information acquisition. Below we also refer to such an output range as a *functional* output range.

## Results

### The models

We studied multiple models that represent either different fundamental steps of a signaling pathway or a complete pathway. All these models include an input or signal molecule *S* and an output *O* that conveys information about the signal’s value. We quantified noise as the average Fano factor (i.e., the variance divided by the mean) of the response distributions. We quantified the output range as the maximal difference of the means of the response distributions. And we quantified information as the mutual information between signal and output (see “Methods”). We estimated these quantities through at least 1000 stochastic simulations for each of *n* evenly distributed values of the number of signal molecules (*N*_*S*_) within the interval [*N*_*Smax*_/*n*,*N*_*Smax*_].

In all our models, the signal is detected by reversibly binding of a molecule to either a receptor *R* or to a DNA binding site (*DNA*_*bs*_). Hence, all models contain at least one reversible binding interaction between molecules. We describe the affinity of two reversibly binding molecules with the equilibrium constant *K*_*eq*_(M) = *k*_*d*_/*k*_*a*_, where *k*_*d*_ and *k*_*a*_ represent the dissociation and association rate, respectively. The equilibrium constant represents the concentration of free signal molecules at which half of the receptors are bound to a signal molecule. As the equilibrium constant decreases, the concentration of signal molecules required to occupy 50% of the receptors decreases too. Hence, smaller *K*_*eq*_ means stronger affinity.

Throughout this paper, we will refer to weak, intermediate and strong affinities in the following sense. Weak affinity refers to an equilibrium constant that is higher than the maximal concentration of the signal. Strong affinity refers to an equilibrium constant that is lower than the minimal concentration of the signal. Finally, an intermediate affinity refers to an equilibrium constant that is between the minimal and the maximal concentration of the signal. In all our models we considered biologically meaningful values of all biochemical parameters (See “Methods” and Sup Tables 1–5).

### Noise is essential to produce a functional output range for the acquisition of information in reversible binding interactions

**Fig. 2:**
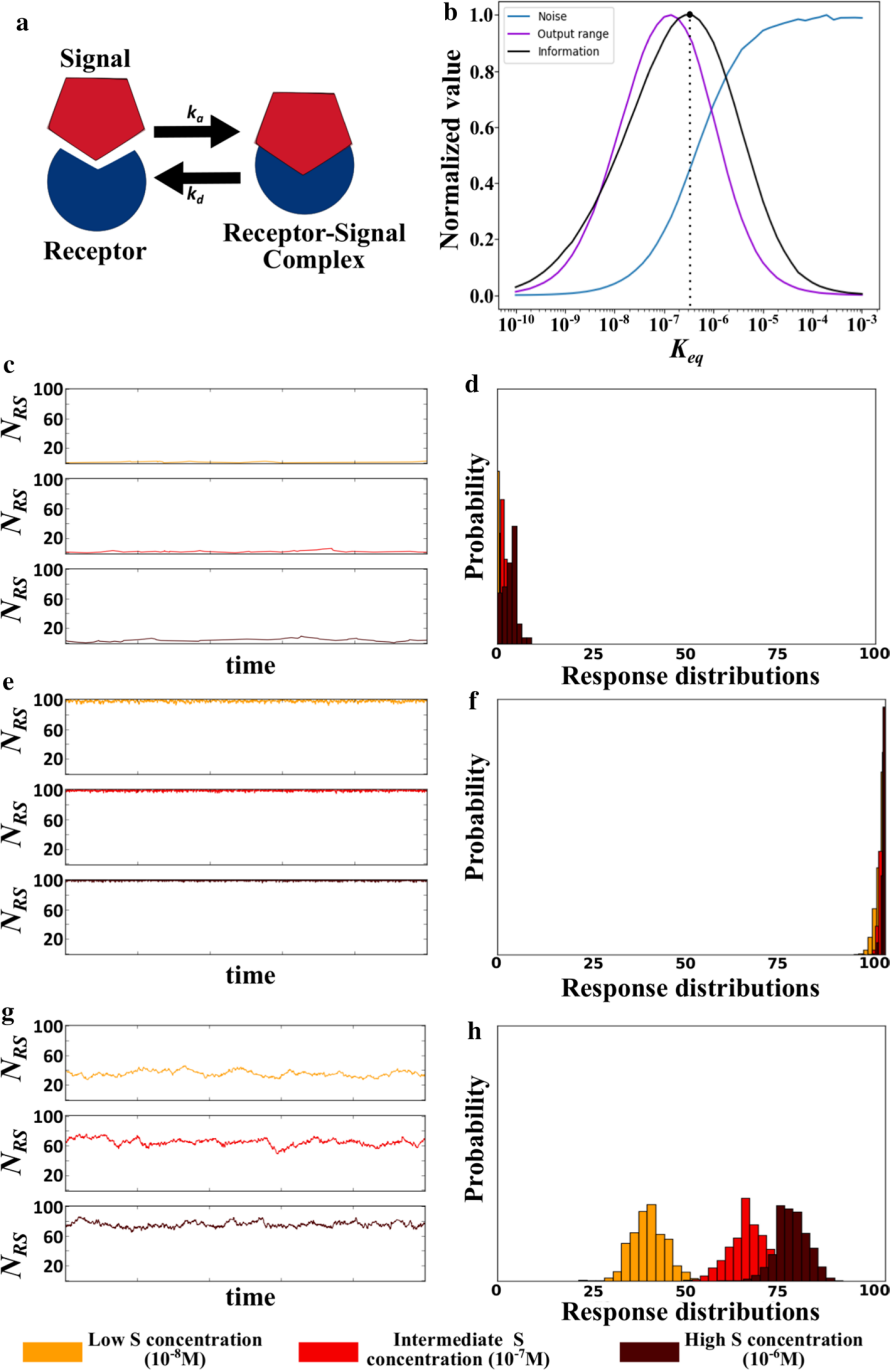
Noise, output range and information in the reversible binding of molecules. **a** Schematic representation of reversible binding involving a receptor and a signal as examples. *k*_*a*_ and *k*_*d*_ correspond to the association and dissociation rate, respectively. **b** Acquired information, output range, and noise for receptor-ligand binding at different affinity values (*K*_*eq*_). The black circle and the dotted line denote the affinity at which the mutual information between signal and output is maximized. Information, noise, and output range are normalized by their respective maximal values. Further panels show the system’s behavior at **c**, **d** weak affinity (*K*_*eq*_ = 10^–5^), **e**, **f** strong affinity (*K*_*eq*_ = 10^–9^), and **g**, **h** intermediate affinity (*K*_*eq*_ = 10^–7^; **g**, **h**). **c**, **e**, **g** show the temporal dynamic of the receptor-signal complexes (N_RS_) at three different concentrations of the signal *S*. **d**, **f**, **h** show response distributions of the number of receptor-signal complexes at these signal concentrations (see color legend at the bottom of the figure)

We first studied the reversible binding between two types of molecules, *S* and *R* that form *RS* complexes (Fig. 2a). In this highly simplified model of an information transmission system, we considered the number of *RS* complexes as the output or response that conveys information about the presence of the signal *S*. Although this notation is suggestive of interactions between a signal (*S*) and a receptor (*R*), our framework below applies to any other reversible binding of two molecules that form a complex. However, for simplicity, we will refer to *R* molecules as receptors, and to *S* molecules as signal molecules.

We asked how noise, output range and information change with the affinity between receptor and signal molecules. For this analysis, we assumed that the concentration of receptors is 10^−8^ M and that the concentration of signal molecules lies within the range [10^−8^ M, 10^−6^ M]. This means that the maximal number of signal molecules is greater than the total number of receptors. Our simulations allow us to distinguish three regimes as a function of affinity. First, when affinity is weak, noise is maximal, and the output range is close to zero (Fig. 2b). Noise is maximal, because even though the small fluctuations in the number of receptor-signal molecules (Fig. 2c) cause a small variance, the mean number of receptor-signal complexes is also always close to zero (Additional file 1: Sup Fig. 1). Conversely, the output range is close to zero, because the number of receptor-signal complexes (*N*_*RS*_) is small for all values of the signal concentration (i.e., *N*_*RS*_ → 0 for all *N*_*S*_ as *K*_*eq*_ → ∞; Fig. 2c). Due to the small output range, response distributions overlap greatly (Fig. 2d), causing information to approach zero (Fig. 2b).

Second, when affinity is strong, receptors are saturated at most or all signal concentrations (Fig. 2e). Consequently, the mean number of receptor-signal complexes is maximal (Additional file 1: Sup Fig. 1), while their fluctuations are minimal (Fig. 2e), resulting in a small variance (Additional file 1: Sup Fig. 1). Thus, noise approaches zero (Fig. 2b). The output range also approaches zero (Fig. 2b), because the number of receptor-signal complexes barely fluctuates from its large value, such that the number of receptor-signal complexes is equal to the total number of receptors (i.e., *N*_*RS*_ → *N*_*R*_ for all *N*_*S*_ as *K*_*eq*_ → 0; Fig. 2e). Again, due to the small output range, the overlap between response distributions is large (Fig. 2f), and acquired information approaches zero (Fig. 2b).

All this changes at intermediate affinities, where receptors can acquire information about the number of signal molecules, because receptors are no longer mainly saturated or unoccupied. Instead, the number of receptor-signal complexes fluctuates (Fig. 2g), increasing the variance (Additional file 1: Sup Fig. 1). At the same time, because receptors are not saturated (Fig. 2g), the mean number of receptor-signal complexes decreases (Additional file 1: Sup Fig. 1). As a result, the level of noise increases (Fig. 2b), but this noise also permits the number of receptor-signal complexes to differ for different number of signal molecules (Fig. 2g). As a result, the output range increases (Fig. 2b), which decreases the overlap between output distributions (Fig. 2h), increasing the acquired information (Fig. 2b). These observations show that noisy signal-receptor binding can be beneficial when a receptor is to acquire information about a signal. In fact, the amount of acquired information is maximal when the level of noise is close to half of its maximal value (Fig. 2b). To verify that our results are not an artifact of numerical simulations, we also solved our model analytically, which yields the same results (Additional file 1: Supplementary text 1 and Sup Fig. 2). In sum, if information is acquired through reversible binding interactions, binding kinetics that yield non-minimal levels of noise are necessary to produce a functional output range, i.e., a response distribution that depends on signal intensity.

### Noise is necessary for information acquisition at realistic ratios of receptor and ligand concentrations

**Fig. 3:**
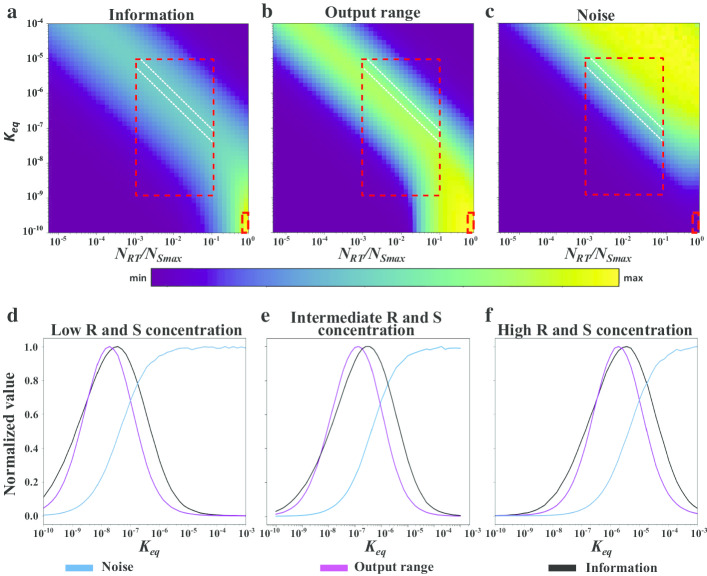
Acquired information, output range and noise at different signal concentrations. Contour plots of **a** information, **b** output range, and **c** noise at different receptor signal ratios *N*_*RT*_/*N*_*Smax*_ (*x* axis), and at different affinities (*y* axis). **a**–**c** The diagonal white-dashed lines circumscribe the region with maximal information acquisition. The large red-dashed rectangles circumscribe the region with biologically observed ranges of receptor-signal affinities ([10^−6^ M, 10^−9^ M]) and *N*_*RT*_/*N*_*Smax*_ ratios (*N*_*RT*_ = 10^–8^ and 10^−7^ M ≤ *N*_*Smax*_ ≤ 10^−5^ M). The small red-dashed rectangles circumscribe the region where *N*_*Smax*_ = *N*_*RT*_ and where the system is noise-free, reaches the maximally possible output range, and where information acquisition is ‘perfect’. Acquired information, output range and noise are plotted from minimally to maximally observed values, color-coded as indicated by the color bar. **d**–**f** Noise, output range and information observed in numerical simulations of the receptor-signal system at different affinities (K_eq_) and with different concentrations of *R* and *S*. **a** *R* = 10^−9^ M and *S* = [10^−9^ M, 10^−7^ M]. **b** *R* = 10^−8^ M and *S* = [10^−8^ M, 10^−6^ M]. **a** *R* = 10^−7^ M and *S* = [10^−7^ M, 10^−5^ M]. Information, noise, and output range are normalized by their respective maximal values

Next, we asked if changes in signal and receptor concentrations could also affect information, noise, and output range. These concentrations, together with the affinity, completely determine the system’s behavior. We varied these concentrations in two different ways. First, we varied the concentrations of the receptors and signal molecules by identical amounts, which keeps the ratio of receptors to signal molecules constant. Second, we only varied the concentration of the signal, which changes this ratio. In both cases, we found the same qualitative relationship between noise, output range, and information as before, as long as the maximal number of signal molecules is in excess of the number of receptors. In other words, efficient information acquisition requires a high output range, which is only produced when the system has a non-minimal level of noise (Fig. 3). Information acquisition is optimal when noise is close to half its maximal value (Fig. 3a–c; white diagonal lines). The higher the signal concentrations are, the weaker are the affinity values required for efficient information acquisition (Fig. 3d–f). The reason is that a receptor’s affinity to its signal needs to decrease as signal concentration increases, otherwise receptors become saturated and no longer detect signal changes effectively.

The only scenario where low noise allows maximal information acquisition requires fewer signal molecules than receptors (Fig. 3a–c, lower right corners small red rectangle). As an extreme case, one can think of a system with an infinitely large number of receptors, a finite number of signal molecules, and extremely strong receptor-signal affinity. In such a system all signal molecules are bound to receptors. Because there are fewer signal molecules than receptors, the system effectively ‘counts’ the number of signal molecules through the number of receptor-signal complexes. Notice that experimentally measured affinity values between receptors and signals, are not extremely strong. Instead, they are of the same order of magnitude as signal and receptor concentrations [11, 33]. In our simulations, these are the affinity values where high information acquisition entails non-minimal levels of noise (Fig. 3a–c big red rectangle), suggesting that biological systems operate in the noisy regime. In sum, under biologically feasible conditions, a functional output range for information acquisition is only produced in the presence of noise.

### Noise is also necessary for information acquisition in consecutive reversible binding interactions

In a signaling pathway, the binding of a signal to a receptor is usually the first of a chain of reversible events. These events include the reversible modification of one or more intermediary signaling molecules, and they usually terminate in the reversible binding of transcriptional regulators, such as a transcription factor (TF), to DNA. TF-DNA binding differs from other signaling binding interactions because most regulated genes exist at one or few copies in any one genome, and any one regulated gene harbors few—usually fewer than ten—TF-binding sites [34, 35]. In the simplest signaling pathways, signal-bound receptors can directly regulate transcription without intervening signaling steps [36].

To study how TF-DNA binding might affect information acquisition in such a pathway, we model two consecutive reversible binding interactions. They represent the formation of a receptor-signal complex, and the binding of this complex to a DNA binding site (*DNA*_*bs*_). We assumed that the concentration of receptor molecules is 10^−8^ M, that the concentration of signal molecules lies in the interval [10^−8^ M, 10^−6^ M], and that a single DNA binding site mediates transcriptional regulation. We view the receptor-signal-*DNA*_*bs*_ complex as the ultimate system output that harbors information about the signal.

We analyzed how the affinities of both the receptor to the signal (*K*_*eqR,S*_) and of the receptor-signal complex to DNA (*K*_*eqRS,D*_) affect the acquisition of information, output range and noise. As in the simpler two-molecule system, the receptor is able to detect different signal concentrations at intermediary receptor-signal affinity, where the largest output ranges are produced with non-minimal levels of noise (Additional file 1: Sup Fig. 3 ).

**Fig. 4:**
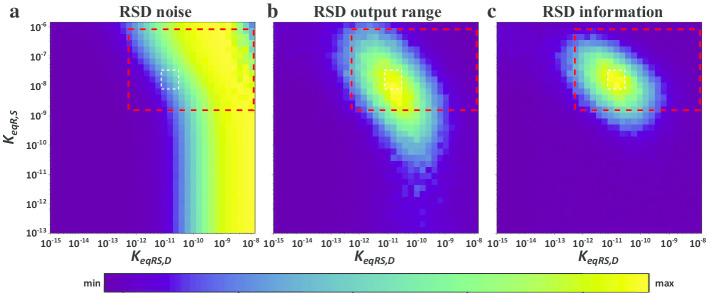
Information, output range and noise in a pair of reversible binding interactions. Contour plots of **a** noise, **b** output range, and **c** information acquisition in the receptor-signal-*DNA*_*bs*_ complex (RSD) as a function of the affinities between both the receptor and the signal (*K*_*eqR,S*_), and the receptor-signal complex with the downstream molecule (*K*_*eqRS,D*_). Red-dashed rectangles circumscribe biologically sensible receptor-signal DNA affinities ([10^−8^ M, 10^−13^ M]) and receptor signal affinities ([10^−6^ M, 10^−9^ M]). White-dashed rectangles delineate the region of maximal information acquisition at the receptor-signal-*DNA*_*bs*_ level. Acquired information, output range and noise are plotted from minimally to maximally observed values, color-coded as indicated by the color bar

To subsequently transmit the information acquired by the receptor-signal complex to the receptor-signal-*DNA*_*bs*_ complex, DNA binding needs to be subject to the same kind of behavior. Noise increases at intermediary affinity values in receptor-signal-DNA binding (Fig. 4a), but this increase also leads to different probabilities of DNA binding for different concentrations of the receptor-signal complex, which increases the output range (Fig. 4b). As a result, the acquisition of information increases (Fig. 4c). In sum, information about a signal is obtained at intermediate values of both affinities (compare the white rectangles, indicating the region with maximal information at the receptor-signal-*DNA*_*bs*_ level in Fig. 4). We also note that the affinities leading to high information acquisition and high noise in our model are similar to experimentally measured affinities between receptors and signals, as well as between transcriptional regulators to DNA (Fig. 4, large red rectangles). Repeating our analysis with up to ten DNA binding sites leads to the same conclusion (Additional file 1: Sup Fig. 4): A noisy dynamic is essential to acquire information.

### Gene expression regulation also requires a noisy dynamic to acquire information

**Fig. 5:**
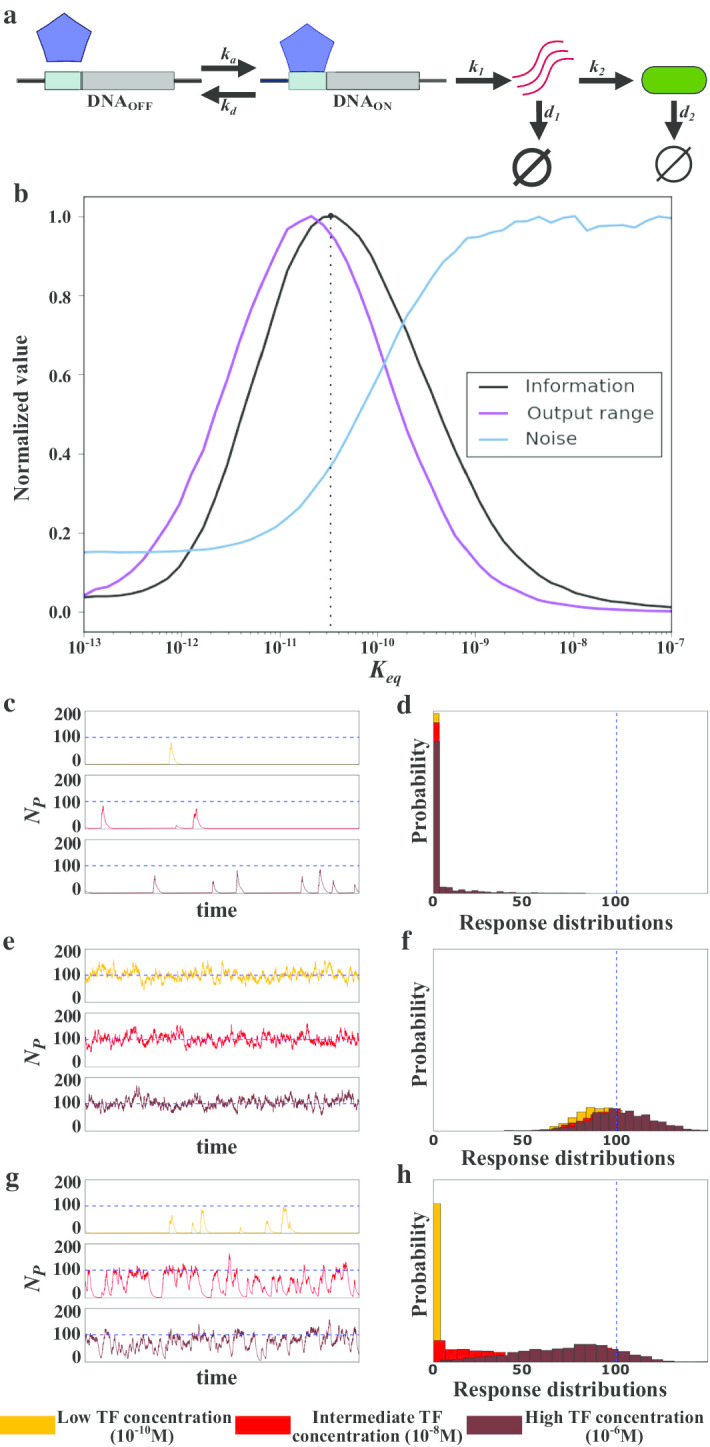
Noise, output range and information in gene regulation. **a** Schematic representation of our model of gene regulation. *k*_*a*_ and *k*_*d*_ correspond to the association and dissociation rate, respectively of a TF with its DNA binding site; *k*_1_ and *k*_2_ correspond to the mRNA and protein synthesis rate, respectively; *d*_1_ and *d*_2_ correspond to the mRNA and protein degradation rates, respectively. **b** Information, output range, and noise observed in numerical simulations of the system at different TF-DNA affinities (*K*_*eq*_). Information, noise, and output range are normalized by their respective maximal values. The black dotted line denote the affinity at which mutual information between signal and output is maximal. System behavior at weak (*K*_*eq*_ = 10^–9^; **c**, **d**), intermediate (*K*_*eq*_ = 10^–11^; **e**, **f**), and strong (*K*_*eq*_ = 10^–13^; **g**, **h**) affinities. Temporal protein dynamics at three different TF concentrations are shown in **c**, **e** and **g**. Response distribution of the number of protein molecules for the same simulations are shown in **d**, **f** and **h**. The blue dashed line in **c**–**h** marks the expected mean protein value for constitutive (unregulated, always-on) expression

At the end of signaling pathways stands the regulation of gene expression, which usually requires reversible binding of a transcription factor to DNA, and additionally involves the synthesis and degradation of mRNA and protein. To find out whether the observed relationship between information, output range, and noise is similar in the presence of such synthesis and degradation, we modeled the regulation of gene expression mediated by a transcription factor that reversibly binds to DNA. We assumed that a gene with a single DNA binding site drives transcription initiation, which occurs only when the binding site is bound by a transcription factor. In this case, mRNA molecules are transcribed at rate *k*_1_, and proteins are translated from the mRNA molecules at a rate *k*_2_. Both mRNA and protein molecules become degraded at rates, *d*_1_ and *d*_2_, respectively (Fig. 5a). We considered the number of TF molecules as the signal, and the number of protein molecules *N*_*P*_ as the output or response.

We started by analyzing how a TF’s affinity to its *DNA*_*bs*_ affects the relationships between information, output range, and noise (Fig. 5b). As in receptor-signal binding, at the weakest affinities, the DNA binding site is almost never bound by TF molecules, regardless of the number of TF molecules (Additional file 1: Sup Fig. 5a). Hence, little mRNA and protein is produced, independently of the number of TF molecules (Fig. 5c and Additional file 1: Sup Fig. 5), producing little variation in the output. Nevertheless, noise is high because the mean number of mRNA and proteins is close to zero (Fig. 5b, c). Because the response distributions are similar, independently of the TF concentration, with a mean close to zero (Fig. 5b, d), the output range approaches zero as well. As a result, as the affinity approaches zero, so does the acquired information (Fig. 5b).

At the strongest affinities, noise shows one noticeable difference to the reversible binding of molecules (Fig. 2b): it does not decrease to zero (Fig. 5b). The reason is that mRNA and protein production have ‘bursty’ dynamics with large excursions from a base line. This bursty dynamics comes from the stochastic nature of mRNA and protein production, which causes fluctuations in the concentration of both kinds of molecules [37]. For this reason, gene expression is intrinsically noisy. In particular, for a gene with constitutive expression, the expected number of protein molecules *N*_*P*_ and its expected noise (standard deviation) are equal to $E({N}_{P})=({k}_{1}/{d}_{2})({k}_{2}/{d}_{1})$ and $E(\sigma ({N}_{P}))=\sqrt{(k1/d2){(k2/d1)}^{2}}$, respectively [38]. At the strongest affinities, our system behaves like a constitutive gene, because TF molecules are almost always bound to the *DNA*_*bs*_ (Additional file 1: Sup Fig. 5a), and the regulated gene is thus almost always transcribed. Accordingly, in our simulations, the mean number of expressed protein molecules and its standard deviation are close to the expected values for a constitutive gene, independently of the number of TF molecules (Fig. 5e; Additional file 1: Sup Fig. 5c; Sup Fig. 6). Consequently, we observe a modest (non-zero) amount of noise (Fig. 5b). However, because the response distributions are similar for all number of TF molecules (Fig. 5f), and the output range tends to zero (Fig. 5b), the amount of acquired information is small (Fig. 5b).

At intermediary affinities, the DNA binding site is not always bound by a TF. It fluctuates between a bound (active) state, when protein molecules are synthesized, and an unbound (inactive) state, when previously synthesized proteins are degraded (Fig. 5g and Additional file 1: Sup Fig. 5a). These fluctuations increase the variance of the response distribution, which increases noise in protein concentrations relative to weak and strong affinities (Fig. 5b). They also increase the output range of the system (Fig. 5b). Most importantly, the probability that a binding site is bound by a TF changes with the number of TF molecules, which renders the system’s output—the number of synthesized proteins—sensitive to its input (Fig. 5g). Hence, the amount of information acquired about this input increases too (Fig. 5b). These observations hold independently of the synthesis and degradation rate of mRNA and protein molecules (e.g., Additional file 1: Sup Fig. 7).

Next we wondered whether the relationship between noise, output range and information at the gene expression level changes in a complete signaling pathway. To find out, we assembled all of the systems discussed above—receptor-signal, TF-DNA, and gene regulation—into a model of a simple complete pathway (see Additional file 1: Supplementary text 2). This pathway is akin to a nuclear hormone receptor pathway, such as the signaling pathway of estrogen, progesterone, and various other lipid-soluble signals [36]. In this pathway, we quantified the amount of information about the concentration of the input (hormone) signal that is contained in the number of expressed protein molecules. This analysis confirmed our previous results. As in the simpler systems, maximal information acquisition requires noise, which increases a pathway’s sensitivity to variation in the signal (Additional file 1: Sup Fig. 8). In other words, noise is also necessary for a functional output range of gene expression, and thus for information acquisition in a complete signaling pathway.

### A noisy dynamic is also required for information acquisition when extrinsic noise is present

Our models thus far focused on intrinsic noise, i.e., noise which affects only some molecules or genes. For example, a TF’s stochastic binding to a gene’s promoter does not affect all genes but only the regulated gene. However, gene expression is also affected by extrinsic noise, i.e., by fluctuations in global properties such as temperature or the number of RNA polymerase molecules, which affect the expression of all genes [37]. We next explored the effect of extrinsic noise on the relationship between the noise of the output, the output range, and acquired information.

To this end, we focused on our gene regulation model, in which we included a second reversible DNA binding interaction to simulate polymerase binding (see “Methods”). The binding of two types of molecules can affect the acquisition of information [39]. We assumed that polymerase affinity to DNA is 10^–9^ (M), a realistic value based on empirical data [40]. To explore the effect of extrinsic noise, we selected a different number of polymerase molecules for each simulation. Polymerases, and housekeeping genes in general, are highly expressed and have low levels of noise [41]. With this observation in mind, we modeled extrinsic noise by randomly selecting the polymerase concentration for each simulation from a negative binomial distribution with a mean 10 times higher than the maximal number of TF molecules (Sup Table 5). We explored three different levels of extrinsic noise by selecting the number of polymerase molecules from a negative binomial distribution with the three different standard deviations, i.e., 0%, 10% and 30% of the mean. Our simulations show that extrinsic noise through polymerase-DNA binding decreases the acquisition of information. However, the relationship between the noise of the output, output range and information persists (Additional file 1: Sup Fig. 9). In sum, our analysis shows that extrinsic noise in polymerase concentrations leaves the qualitative relationship between noise, output range and information unchanged (Additional file 1: Sup Fig. 9).

## Discussion

A fundamental step in signaling pathways is the reversible binding of molecules, which is necessary for the detection of a signal by receptors and for the acquisition of information about this signal. Previous experimental and theoretical work has demonstrated that biological processes, including signaling pathways and their binding interactions, are inherently noisy [37]. Here, we find that reversible binding in signaling pathways noise is indispensable to increase a pathway’s output range. Increasing the output range is necessary to produce different responses to different stimuli. In other words, noise is essential to acquire information.

While noise is necessary to acquire information, the maxima of acquired information and noise occur at different affinity values, because they depend in different ways on molecular affinities. Information requires an output range that reflects different responses to different stimuli. This output range is produced, for example, when a receptor can detect a signal, which is possible when the receptor is sensitive to changes in the signal, and not saturated by the signaling molecule. This happens at intermediary affinities, when the signaling molecule can readily bind and unbind the receptor. Conversely, noise is maximal at weak affinities, because that is where binding events are rare and produce low mean (receptor-ligand) concentrations. These low mean concentrations result in a small output range that discriminates poorly between different stimuli.

Noise can have multiple benefits for biological processes. These benefits include the production of phenotypic heterogeneity [42–48], the emergence of patterns in morphogenesis [49, 50], efficient synchronization of biological oscillations [51–55], and various evolutionary advantages [15, 56–60]. Moreover, noise can also benefit the acquisition of information. For example, in bistable systems, a weak signal might not be able to push the system into a different state unless it exceeds a detection threshold. In this case, noise can produce fluctuations that helps improve a system’s sensitivity to weak signals and allows it to switch to a different state, a phenomenon known as stochastic resonance [18–20, 22]. Our results go beyond those predicted by stochastic resonance, because our models are not bistable. A signal thus does not need to pass any threshold to switch system behavior. Instead, we find that the importance of noise for signaling simply emerges from reversible binding interactions. In such interactions, noise is absent only when molecules bind each other permanently or are unable to bind, in which case their interactions cannot respond to environmental changes, and can thus not transmit information during signaling. In other words, information transmission requires noisy binding dynamics even in the absence of signal detection thresholds.

There is only one condition—very strong signal-receptor affinity—where noise is not required for information acquisition through reversible binding interactions. Under this condition, a noise-free ‘perfect’ detection of a signal is possible when the number of receptors is greater than the number of signal molecules. However, producing more receptors than signaling molecules would incur enormous energetic costs. Relatedly, transcriptional regulation generally involves fewer than ten TF binding sites per regulated gene—the analog of a receptor in such a system [34, 35]—a number that is much smaller than the average number of transcription factors per cell, which are usually in the hundreds for bacteria and in the thousands for yeast and mammal cells [61, 62]. Hence, a perfect detection of the number of TFs or signal molecules is not biologically plausible.

Recent information theoretic studies of signaling pathways have found that non-minimal levels of noise facilitate information acquisition [11, 23, 24], but these studies were not ideally suited to understand the mechanisms by which noise helps increase information acquisition. They either did not include a mechanistic description of signaling pathways, they did not model molecular interactions explicitly, or they assumed that noise comes from an external source and can be made arbitrarily small. In contrast, our models represent molecular interactions explicitly, which causes noise to emerge naturally from them. In doing so, they also provide a mechanistic explanation of the relationship between noise and information acquisition.

One limitation of our work is that it focuses on the simplest molecular interactions, and does not exhaust all possible interactions. These include feedback loops and feed forward loops, which modify both noise and information [9–11, 32, 39, 63–65]. They include TF dimerization, which can reduce noise [12, 66–68], and increasing the number of molecules that sense and transmit the presence of a signal, which can increase the output range [69, 70]. In addition, information acquired by an organism can increase without an upper bound when the organism uses multiple independent pathways to detect a signal [9]. As a result of such work, we know that noise—and by implication also information acquisition—can be tuned within some limits [23, 37, 38, 69, 71, 72]. The analysis of such complexities, and how they affect the relationship between noise and information acquisition remains an important task for future work.

Noise at the molecular level can be subdivided into extrinsic and intrinsic noise. Extrinsic noise refers to fluctuations that affect many processes simultaneously. They include fluctuations in temperature, ribosome numbers, and numbers of RNA polymerase molecules. Intrinsic noise refers to fluctuations that affect particular biochemical reactions or the expression of particular genes. One example is variation in the copy number of specific transcription factors [6, 37]. Our results suggest that non-minimal levels of noise are necessary even when both types of noise are considered. However, TF-polymerase-DNA interactions affect noise-related properties that we did not study explicitly, such as the frequency and amplitude of transcription bursts [73, 74]. Extrinsic noise also decreases the acquisition of information. More comprehensive work will be needed to understand how intrinsic and extrinsic noise together affect information acquisition.

Our models include multiple simplifying assumptions. For example, we assumed that the numbers of signaling molecules, receptors, and transcriptional regulators are constant, whereas they may change dynamically in cells. We considered that a signal has a uniform distribution within a given concentration interval, whereas environmental signals may have different (usually unknown) distributions [28]. Signal distribution is important because it defines information theoretic properties such as channel capacity [27]. In addition, we did not consider molecular interactions such as dimerization [66, 67]. Similarly, we did not consider the costs of expressing an information processing machinery [69]. Because these factors do not affect the nature of reversible binding, we suspect that they might also not reduce the positive role of noisy binding dynamics for information acquisition. However, it remains to be seen if such different mechanisms interact and affect our observations.

## Conclusions

Our work shows that the kinetic parameters of signaling pathways must produce noisy binding dynamics or a signaling pathway will acquire little or no information. This is due to the nature of reversible binding interactions. Under biologically sensible parameter values and realistic concentrations of ligands and receptors, binding of molecules is noise-free only when a receptor is completely saturated or permanently bound by its ligand, or if it is unable to bind the ligand. In either case, information acquisition is impossible without a noisy dynamic. Because reversible binding interactions are used by all signaling pathways, noise is not just unavoidable but a necessary condition for information acquisition in signaling pathways.

## Methods

### Reversible and consecutive molecular binding models

We consider two kinds of molecules, *S* (signal) and *R* (receptor), which can associate reversibly into receptor-signal complexes at an association rate *k*_*a*_ (M^−1^ s^−1^), and a dissociation rate *k*_*d*_ (s^−1^).

To model consecutive reversible binding steps, we assume that, first, a signal (*S*) and a receptor (*R*) reversibly associate into a receptor-signal (*RS*) complex. Second, this complex binds reversibly to a downstream molecule (*D*), such as DNA. We denote the rate of association between the signal and the receptor by *k*_*aR,S*_ (M^−1^ s^−1^), and that of dissociation by *k*_*dRS*_ (s^−1^). Similarly, we denote the rate of association between the receptor-signal complex and the downstream molecule by *k*_*aRS,D*_ (M^−1^ s^−1^), and that of dissociation by *k*_*dRSD*_ (s^−1^).

### Gene expression system

We model a gene expression system where one chemical species, denoted as *TF* (transcription factor), binds to a DNA binding site (*DNA*_*bs*_) to regulate the expression of a nearby gene. *TF* molecules associate with the *DNA*_*bs*_ at a rate *k*_*a*_ (M^−1^ s^−1^). The dissociation of TF-*DNA*_*bs*_ complexes happens at a rate *k*_*d*_ (s^−1^). In the disassociated state, no transcription occurs, and in the associated state transcription occurs at a rate *k*_1_ (s^−1^). Transcribed mRNA molecules are degraded at a rate *d*_1_ (s^−1^). Finally, proteins are translated from mRNA molecules at a rate *k*_2_ (s^−1^), and degraded at a rate *d*_2_ (s^−1^).

### Gene expression system with extrinsic noise

We model an extended version of the gene expression system with a polymerase. In this model, the polymerase is only able to bind DNA that is already bound by a TF molecule. This avoids leaky gene expression and simplifies the model. We assume that polymerase molecules (P) bind to TF-*DNA*_*bs*_ complexes at a rate *k*_*aTF-DNA,P*_ (M^−1^ s^−1^). TF-P-*DNA*_*bs*_ complexes dissociate into TF-*DNA*_*bs*_ + P at rate *k*_*dTF-P-DNA*_ (s^−1^), and they dissociate into TF + *DNA*_*bs*_ + P at rate *k*_*d*_ (s^−1^). The latter rate is also the dissociation rate of TF-*DNA*_*bs*_ complexes, because it represents the unbinding of P molecules from DNA caused by the unbinding of the TF to DNA. To model extrinsic noise, we selected for every simulation a different number of polymerase molecules from a negative binomial distribution with a mean value 10 times of the maximal number of TF molecules, and a standard deviation of 0% (no extrinsic noise), 10% or 30%, to explore different levels of extrinsic noise.

### Complete linear signaling pathway

Our model considers the reversible receptor-ligand complex formation and gene expression activation, which is mediated by the receptor-signal complex. Consequently, the parameters that govern the behavior of such a pathway are similar to those described so far, namely: (1) an association rate (*k*_*aR,S*_) between the signal and receptor (*R*) and a dissociation rate(*k*_*dRS*_) of the receptor-signal complexes (*RS*), (2) an association (*k*_*aRS,D*_) and a dissociation (*k*_*dRSD*_) rate between *RS* and a DNA binding site (*DNA*_*bs*_), and (3) a rate of gene transcription (mRNA synthesis, *k*_1_), mRNA degradation (*d*_1_), protein synthesis (*k*_2_), and protein degradation (*d*_2_).

### Stochastic simulations

We simulated the behavior of the models described above using Gillespie’s discrete stochastic simulation algorithm [75], using the numpy python package for scientific computing (https://www.numpy.org/). Gillespie’s algorithm captures the stochastic nature of chemical systems. It assumes a well-stirred and thermally equilibrated system with constant volume and temperature. The algorithm requires the propensity *p*_*j*_ that a chemical reaction *R*_*j*_ occurs in a given time interval [t,t + τ). Any such propensity *p*_*j*_ is proportional to both the reaction rate and the number of reacting molecules. Notice that for first-order reactions, such as the dissociation of a molecular complex into its constituent molecules, *p*_*j*_ is independent of the volume in which the reaction takes place. In contrast, *p*_*j*_ is inversely proportional to the volume in second-order reactions, such as the association of two molecules. For the reversible molecular binding modeled here, the propensities *p*_*a*_ and *p*_*d*_ that two molecules associate and dissociate, respectively, are proportional to

$$\begin{aligned} p_{a} & = \frac{{k_{a} }}{{VN_{A} }}N_{a} N_{b} \\ p_{d} & = k_{d} N_{c} \\ \end{aligned}$$

where *V* is the reaction volume, *N*_*A*_ is Avogadro’s number, and *N*_*a*_, *N*_*b*_, and *N*_c_ are the numbers of molecules of the two chemical species *a* and *b* and of the complex *c*.

The propensities *p*_*mRs*_, *p*_*mRd*_, *p*_*Ps*_ and *p*_*Pd*_ of a mRNA transcription, mRNA degradation, protein synthesis, and protein degradation event are given by

$$\begin{aligned} p_{{mR_{s} }} & = k_{1} N_{c} \\ p_{{mR_{d} }} & = d_{1} N_{mR} \\ p_{{P_{s} }} & = k_{2} N_{mR} \\ p_{{P_{d} }} & = d_{2} N_{P} , \\ \end{aligned}$$

respectively. In these expressions, *t*he quantities *N*_*mR*_ and *N*_*P*_ are the numbers of mRNA molecules and of protein molecules, respectively. We model a haploid organism with only a single DNA binding site, corresponding to a single regulated gene. In this case, the propensity of mRNA synthesis can be reduced to

$${p}_{{mR}_{s}}={k}_{1}$$

when the binding site is bound by transcription factor (*N*_c_ = 1) and to

$${p}_{{mR}_{s}}=0$$

when the binding site is unbound (*N*_c_ = 0).

### Initial conditions for simulations

To determine the initial conditions of the system, we first calculated the expected number of complexes formed as

$$\hat{N}_{C} = N_{{B_{T} }} \frac{{N_{A} }}{{K_{eq} + N_{A} }}$$

The quantity *N*_*A*_ is the number of signal or TF molecules and *N*_*BT*_ is the total number of receptor molecules or DNA binding sites. Notice that $\hat{N}_{C}$ is a real number, and for the specific case of the TF-*DNA*_*bs*_ interaction it can only take a value between 0 and 1. Thus, $\hat{N}_{C}$ equals to the probability that the DNA binding site is bound by a transcription factor. However, for the receptor signal complexes, it represents the number of complexes formed. We selected the initial state of the number of complexes (${{N}_{C}}_{i}$) at random with binomial probability for the binding site. However, we selected the closest integer to ${\widehat{N}}_{C}$ for the receptor signal case. Then we defined

$$\begin{aligned} N_{Ai} & = N_{A} - N_{Ci} \\ N_{Bi} & = N_{{B_{T} }} - N_{Ci} \\ \end{aligned}$$

as the initial state of the number of free signal or TF molecules (${{N}_{A}}_{i}$) and of the receptors or binding sites (${{N}_{B}}_{i}$). Finally, as the initial state of the number of mRNA and protein molecules we used

$$\begin{aligned} N_{{mR_{i} }} & = \hat{N}_{C} \frac{{k_{1} }}{{d_{1} }} \\ N_{{P_{i} }} & = \hat{N}_{C} \frac{{k_{1} }}{{d_{1} }}\frac{{k_{2} }}{{d_{2} }} \\ \end{aligned}$$

which is the expected average number of mRNA and protein molecules for a constitutively expressed gene [37], multiplied by the probability that the DNA binding site is bound by a TF molecule.

### Information quantification

The number of molecules of any chemical species in a cell or in a unit volume fluctuates, because molecules are produced and decay stochastically, and because they undergo random Brownian motion caused by thermal vibrations. We use Shannon’s entropy to quantify the unpredictability caused by such stochastic fluctuations in a signal as

$$H\left(\mathrm{Pr}(S)\right)=-\sum_{{N}_{S}=\frac{{N}_{Smax}}{n}}^{{N}_{Smax}}p({N}_{S}){\mathrm{log}}_{2}p({N}_{S}),$$

where Pr(S) is the probability distribution of the signal, and *p(N*_*S*_*)* is the probability that the system contains *N* molecules of the signal*.* In our models the signal represents either a molecular signal or cue [2].

For all our analyses, we performed at least 1000 simulations for each of *n* different numbers of signal molecules that were evenly distributed within the interval [*N*_*Smax*_/*n*,*N*_*Smax*_] (*n ≤ N*_*Smax*_). For this reason

$$H(\mathrm{Pr}(S))={\mathrm{log}}_{2}n$$

Signals trigger changes in a cell’s state that produce a response or output (*O*) of the system, such as the production of molecules. A cell acquires information when the output *O* reflects (fully or partially) the value of *S*. This information can be quantified via the mutual information:

$$I\left( {S;O} \right) = H\left( {{\Pr}\left( S \right)} \right) - H\left( {{\Pr}(SO)} \right),$$

which is equal to the entropy *H*(Pr(*S*)) minus the conditional entropy *H*(Pr(*S*|*O*)), which represents the entropy of *S* given that *O* is known [27]. In other words, the mutual information *I* quantifies the acquired information as the amount of information that an output chemical species *O* harbors about a signal *S.*

### Noise quantification and output range

The systems we model produce a probabilistic response for any given quantity *N*_*S*_ of the signal. This response can thus be represented as a conditional probability distribution:

$$\Pr(0<N_{O}<N_{O_{max}}|S=N_{S})$$

where *N*_*O*_ and *N*_*Omax*_ are the number and maximal number of output molecules, respectively. We estimated this response distribution through at least 1000 replicate simulations of the system for each value of *N*_*S*_. We then quantified noise as the Fano factor, which is the variance divided by the mean of the response distributions, averaged over all *n* possible values of *N*_*S*_:

$${\overline{\sigma }} = \frac{1}{n}\mathop \sum \limits_{{N_{S} = N_{Smin} }}^{{N_{Smax} }} \frac{{{\upsigma }^{2} ({\Pr}(O|S = Ns))}}{{\mu ({\Pr}(O|S = Ns))}},$$

We define the output range as the difference between the maximal and minimal mean value of all response distributions.

### Parameter values

Our simulations considered the following biologically sensible parameter ranges. The association and dissociation constants *k*_*a*_ and *k*_*d*_ of reversible complex formation define the equilibrium constant *K*_*eq*_ = *k*_*d*_/*k*_*a*_ (M), which we used in our simulations. The smaller *K*_*eq*_ becomes, the more association becomes favored over dissociation [33]. In particular, for the binding between ligands and (nuclear) receptors, we used values of *K*_*eq*_ within the interval [10^−6^ M, 10^−9^ M], because the micromolar to nanomolar range is common for such complexes [33, 61, 77–79]. For TF-DNA binding, empirical data suggests that usually *K*_*eq*_ < 10^–8^ and can reach picomolar (10^−12^ M) or even smaller values [33, 61, 76–78]. Thus, we used values in the interval [10^−8^ M, 10^−12^ M].

For mRNA, experimentally measured half-lives usually lie in the range of seconds to hours [79–82]. Protein half-lives typically lie between hours and days [81, 83]. Taking all this information into consideration, we chose mRNA half-lives within the interval [1 min, 30 min], and protein half-lives where within [15 min, 3 h]. We assume that the ratio *k*_2_/*k*_1_, which describes the speed of the protein synthesis rate relative to the mRNA synthesis rate, exceeds 1.0 [84]. Because the residence time of transcription factors on DNA lies within seconds to hours [85, 86], we assumed a residence time within this interval [10 s, 2 h].

Finally, we always considered concentrations of molecules to lie within the interval [10^−9^ M, 10^−6^ M], because these are typical concentration of most molecules within a cell or a nucleus [87]. Notice that for some of our simulation we also needed to explore values outside these ranges. Specific parameter values used for each simulation are listed in Sup Tables 1–5.

## Supplementary information

Additional file 1. Supplementary figures 1 to 9, supplementary tables 1 to 5, and supplementary texts 1 and 2.

## Data Availability

All data generated or analysed during this study are included in this published article [and its supplementary information files] or is available from the corresponding author on reasonable request.

## Abbreviations

TF: transcription factor
DNA_bs_: DNA dinging site
K_eq_: equilibrium dissociation constant
S: signal
R: receptor
*N*_*S*_: number of signal molecules
*N*_*R*_: number of receptor molecules
*N*_*RS*_: number of receptor-signal complexes
