# Supplementary material for: Signaling pathways have an inherent need for noise to acquire information
Source: BMC Bioinformatics. 2020 Oct 16;21:462. doi: 10.1186/s12859-020-03778-x (PMC7568421; doi:10.1186/s12859-020-03778-x)
Supplement: Supplementary file 1 — Supplementary figures 1 to 9, supplementary tables 1 to 5, and supplementary texts 1 and 2. [file 12859_2020_3778_MOESM1_ESM.pdf]

Supplementary Information: Signaling pathways have an inherent need for noise to acquire information

Eugenio Azpeitia[1,2,3], Eugenio P. Balanzario[3], Andreas Wagner[1,2,4,*]

Supplementary text 1

We want to compute the mutual information $I(S_0; O)$ of the two random variables S_0, which represents the signal, and O, which represents the output. S_0 is the initial number of signal molecules and takes on n values uniformly distributed in the interval $[N_{Smax}/n, N_{Smax}]$, where N_{Smax} is the maximal number of signal molecules. $O = O_\infty$, where $O_t = S_0 - S_t$ and where S_t is a birth and death Markov process determined by the following set of infinitesimal generators

$$\lim_{h\to 0}\frac{p_{j,j+1}(h)}{h} = \lambda_j = k_d(S_0 - j)$$

$$\lim_{h\to 0}\frac{p_{j,j-1}(h)}{h} = \mu_j = k_a(R_0 - (S_0 - j))$$

$$\lim_{h\to 0}\frac{p_{j,j}(h)}{h} = \lambda_j + \mu_j$$

with $p_{i,j}(h) = P\{S_{t+s} = j \mid S_s = i\}$ for $s \geq 0$. R_0 is an initial condition for the number of receptors, equal to the total number of receptor molecules. The birth and death process S_t depends on the parameters k_a and k_d. Let $m = S_0 - R_0$ be the minimum value of O_t and let $M = S_0 + O_0$ be the maximum value. Notice that by now we have four parameters k_a, k_d, R_0 and O_0 that govern the birth and death process. The process is described by the following set of Kolmogorov forward equations

$$p'_{i,m}(t) = \mu_{m+1}p_{i,m+1}(t) - \lambda_m p_{i,m}(t)$$

$$p'_{i,j}(t) = \mu_{j+1}p_{i,j+1}(t) - (\lambda_j + \mu_j)p_{i,j}(t) + \lambda_{j-1}p_{i,j-1}(t)$$

$$p'_{i,M}(t) = -\mu_M p_{i,M}(t) + \lambda_{M-1}p_{i,M-1}(t)$$

with the initial conditions $p_{i,j}(0) = \delta_{ij}$. If we let $\pi_i = p_{i,j}(\infty)$ and let $t \to \infty$ in the Kolmogorov forward equations, then $p'_{i,j}(\infty) = 0$ and we obtain the following set of algebraic equations

$$\pi_{m+1} = \frac{\lambda_m}{\mu_{m+1}}\pi_m, \qquad \pi_{j+1} = \frac{\lambda_j + \mu_j}{\mu_{j+1}}\pi_j - \frac{\lambda_{j-1}}{\mu_{j+1}}\pi_{j-1}, \qquad \pi_{m+1} = \frac{\lambda_{M-1}}{\mu_M}\pi_{M-1}$$

where λ_j and μ_j are as stated above. These equations can be solved recursively. The set $\{\pi_j: m \le j \le M\}$ determines the probability distribution of S_∞ and hence also of $O_\infty = S - S_\infty$.

It is more expedient to write $\pi_j(S_0)$ instead of π_j, since the above computations depend on the value of S_0. To compute the mutual information $I(S_0; O) = H(\Pr(O_\infty)) - H(\Pr(O_\infty \mid S_0))$, we must determine the marginal distribution of $O = O_\infty$. This marginal distribution is given by

$$P\{O_\infty = S_0 - j\} = \sum_{i-m}^{M} P\{S_\infty = j | S_0 = i\} P\{S_0 = i\} = \frac{1}{n}\sum_{i=m}^{M} \pi_j(S_0 = i)$$

and allows us to compute mutual information, output range, and noise (Sup Fig 2).

Supplementary text 2

In the model of a complete signaling pathway the signal-bound receptor molecules. Receptor-signal complexes act as TFs that associate and dissociate from a regulatory binding site on DNA (DNA_{bs}). Once a receptor-signal-DNA_{bs} complex is formed, mRNA is transcribed at rate k_1, and protein is produced from mRNA molecules at rate k_2. mRNA and protein are degraded at rates d_1 and d_2. $K_{eq_{R,S}}$ and $K_{eq_{RS,D}}$ describe the affinity of the receptor to the signal, and the affinity of the receptor-signal complex to the DNA_{bs}, respectively. This model resembles nuclear receptor signaling pathways, where nuclear receptors directly regulate gene transcription (reviewed in (1)). Although many nuclear receptors act as dimers (e.g., (2)), we consider here for simplicity a receptor that acts as a monomeric transcriptional regulator.

Based on our previous result we expected that both reversible binding interactions included in the model, namely the receptor-signal and the receptor-signal-DNA_{bs} interactions, should acquire and transmit information at intermediary affinities values, where both noise levels and the output range are high. At intermediary affinity values, the binding-unbinding dynamic of the receptor-signal with the DNA_{bs} should amplify the fluctuations in the number of proteins, thus increasing both noise and increasing information transmission. Our simulation shows that this is indeed the case. Information acquisition in this simple signaling model is maximized at intermediary affinity values of both binding interactions (Sup Fig 8).

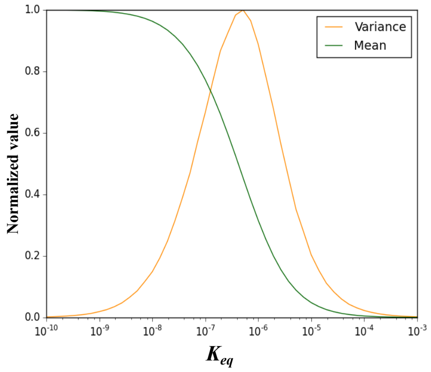

Sup Fig 1. Mean and variance of the number of receptor-signal complexes ($\bar{N}_{RS}$) formed at different affinity values (K_{eq}). The maximum number of receptor-signal complexes for this simulation is 50 (see Sup Table 1). For visualization purposes, mean and variance are normalized by their maximal value.

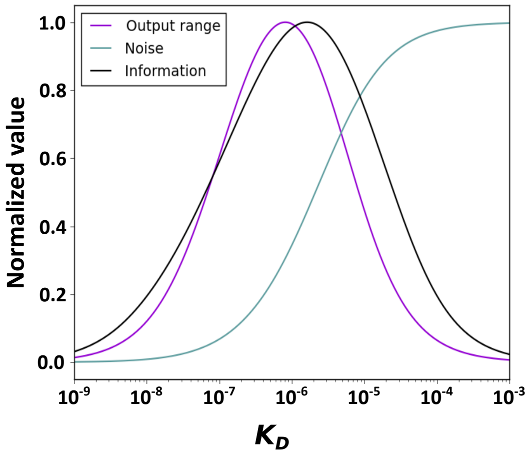

Sup Fig 2. Analytic results of information, noise and output range in the receptor-signal system (see Sup Text 1).

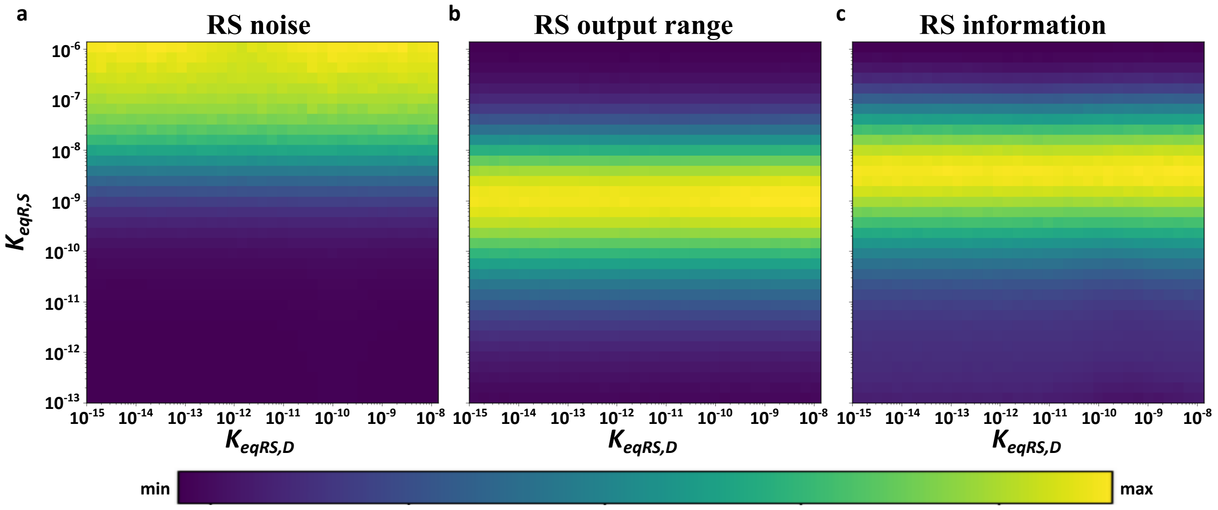

Sup Fig 3. Information, output range and noise in a pair of reversible binding interactions. Contour plots of (a) noise, (b) output range and (c) information acquisition in the receptor-signal complex (RS) as a function of the affinities between both the receptor and the signal ($K_{eqR,S}$), and the receptor-signal complex with the downstream molecule ($K_{eqRS,D}$). Acquired information, output range and noise are plotted from minimally to maximally observed values, color-coded as indicated by the color bar (see Sup Table 2 for the parameters).

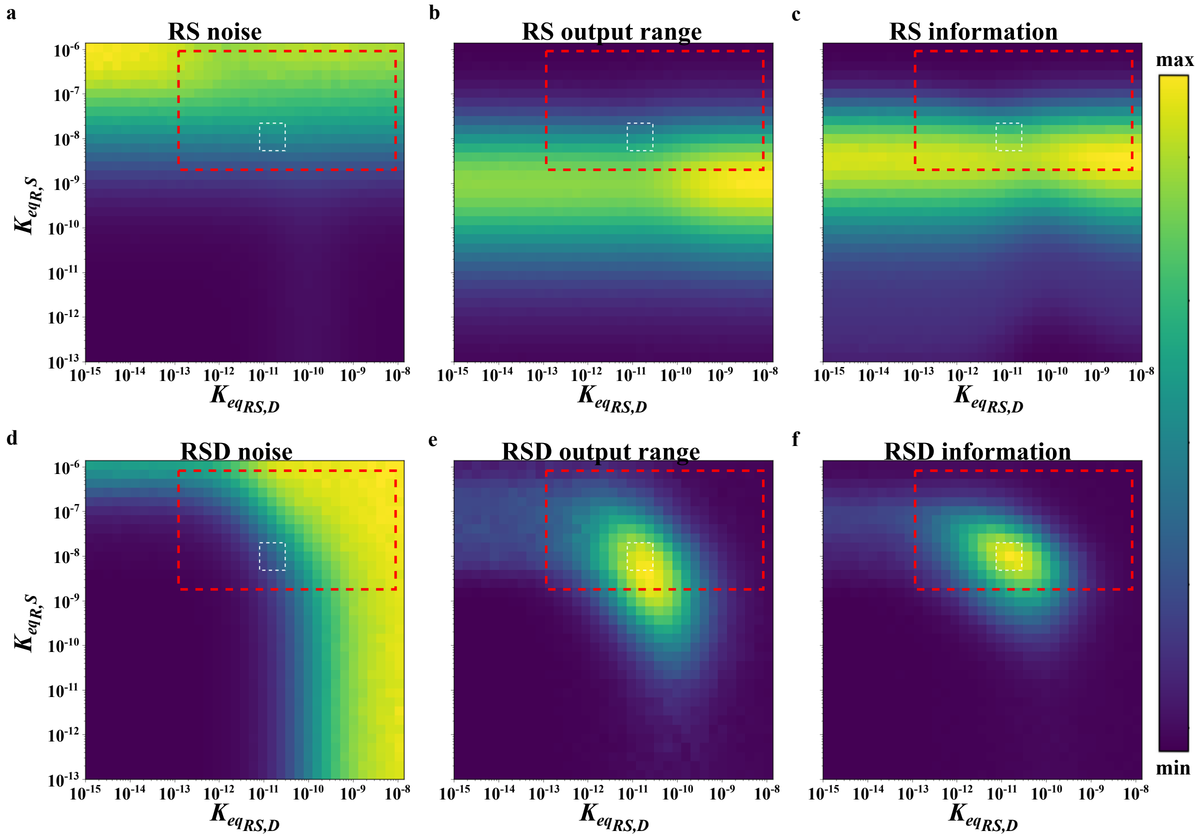

Sup Fig 4. Information, output range and noise in two consecutive binding interactions with ten DNA$_{bs}$. Contour plots of (a and d) noise, output range (b and e) and information acquisition (c and f) in the receptor-signal complex (*RS*; a-c) and in the receptor-signal-DNA_{bs} complex (*RSD*; d-f) as a function of the affinities between both the receptor and the signal ($K_{eqR,S}$), and the receptor-signal complex with the downstream molecule ($K_{eqRS,D}$). Red-dashed rectangles circumscribe biologically sensible receptor-signal DNA affinities ([10^{-8}M,10^{-13}M]) and receptor signal affinities ([10^{-6}M,10^{-9}M]). White-dashed rectangles delineate the region of maximal information acquisition at the receptor-signal-DNA_{bs} level. Acquired information, output range and noise are plotted from minimally to maximally observed values, color-coded as indicated by the color bar (see Sup Table 2 for the parameters).

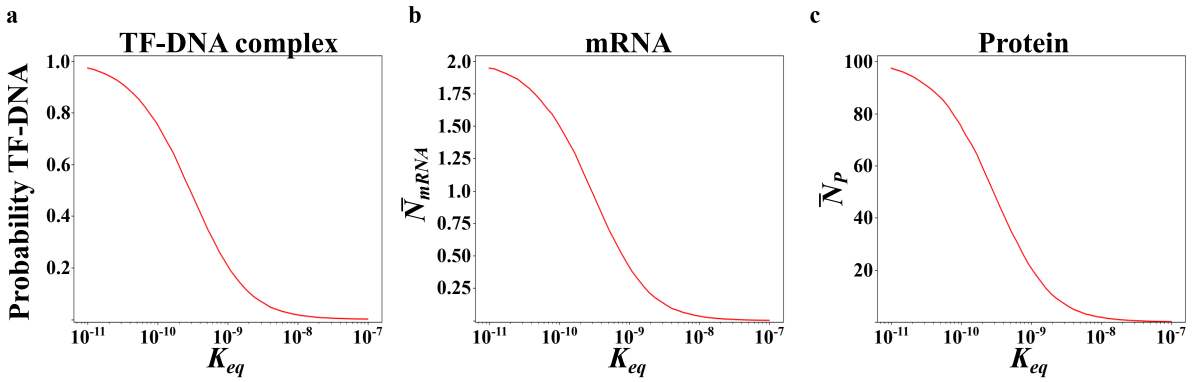

Sup Fig 5. (a) Probability of TF-*DNA* binding, (b) mean number of mRNA molecules ($\bar{N}_{mRNA}$), and (c) mean number of protein molecules ($\bar{N}_P$), as a function of TF-DNA affinity (K_{eq}). For the parameters we used (Sup Table 3), the expected mean number of mRNA and protein molecules produced from a constitutively expressed gene is 2 and 100, respectively.

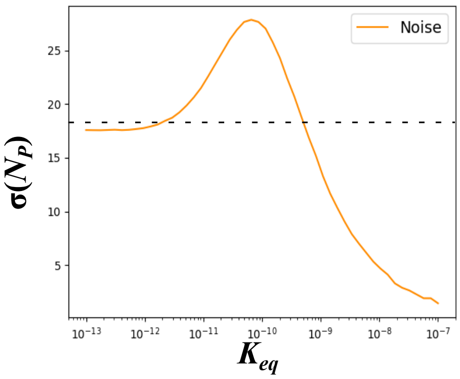

Sup Fig 6. Non-normalized values of standard deviation in the number of proteins. The black line is the standard deviation expected for the parameters used in this simulation (Sup Table 3).

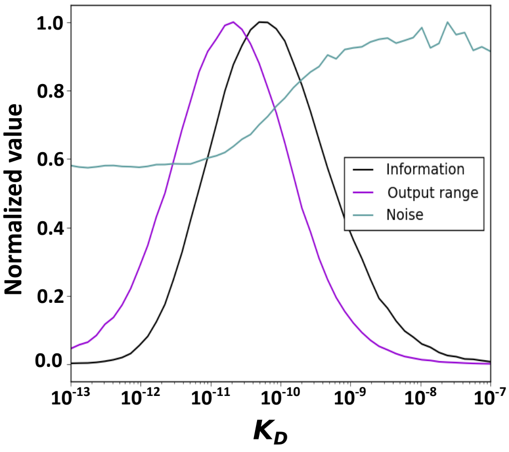

Sup Fig 7. Information, output range and noise in the gene regulation model for proteins with a half-life of ~12h (see Sup Table 3).

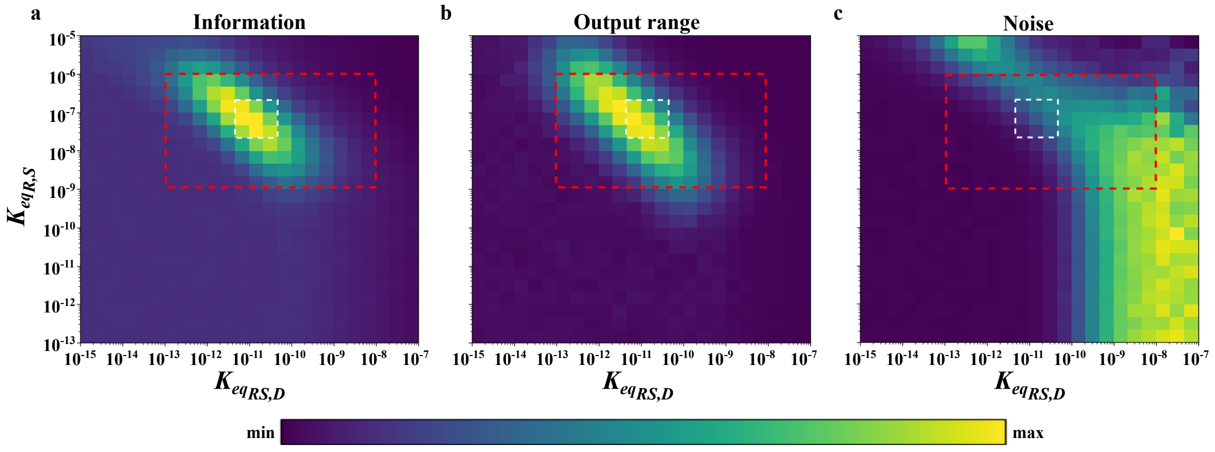

Sup Fig 8. Contour plot of acquired information and noise in a complete signaling pathway. (a) Information, (b) output range, and (c) noise for different affinities between receptor and signal molecules ($K_{eqR,S}$, *y* axis), and receptor-signal complexes with a DNA binding site ($K_{eqRS,D}$, *x* axis) in a model of a simple lineal signaling pathway where a signal bound receptor can bind DNA and regulate gene expression. The number of signal molecules constitute the input into this pathway, and the number of protein molecules expressed from the regulated gene constitute the output. The red-dashed rectangle show experimentally measured affinity values between receptors and signals ([10^{-6}M,10^{-9}M]) and between transcriptional regulators and DNA ([10^{-8}M,10^{-13}M]). The amount of acquired information and noise level are indicated in the color bar (see Sup Table 4 for the parameters).

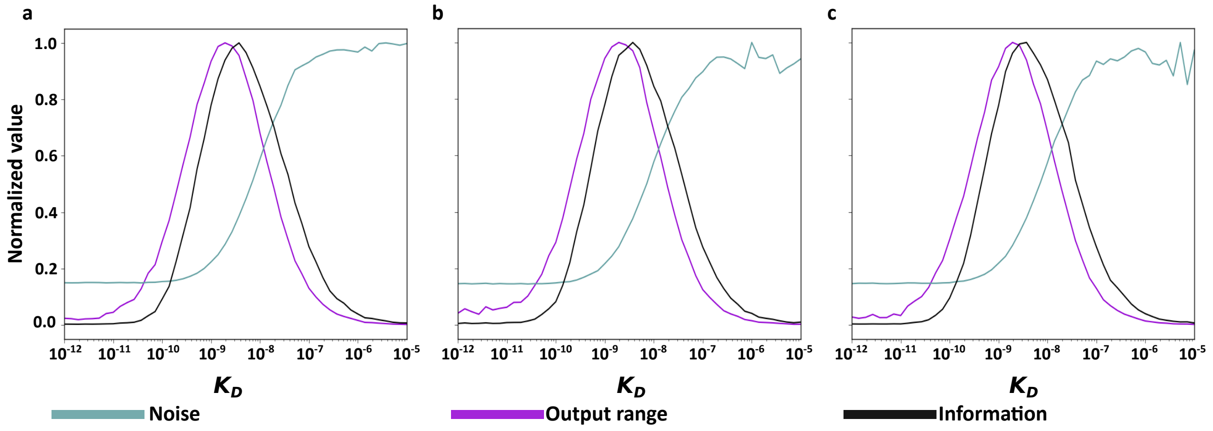

Sup Fig 9. Acquired information, output range, and noise in a gene regulation model that includes the binding of RNA polymerase subject to extrinsic noise. Noise, output range, and information observed in numerical simulations of the gene expression system with added polymerase-DNA binding. The affinity between the polymerase and the TF-DNA is 10^{-9}. The mean number of polymerase molecules is 10 times the maximal number of TF molecules (see N_{Tmax} in Sup Table 5) and has a standard deviation of (a) 0% (no extrinsic noise) (c) 10% and (d) 30%. Information, noise, and output range are normalized by their respective maximal values.

Sup Table 1. Parameter values used for the receptor-signal model.

	k_d (s^{-1})	k_a ($M^{-1}s^{-1}$)	N_{Smax}	N_{RT}	Volume (L)	n
Fig 2 and Sup Fig 1 and 2	0.001	$[10,10^7]$	5000	50	8.3×10^{-15}	50
Fig 3a-c	0.001	$[10^1,10^7]$	[50,5000]	50	8.3×10^{-15}	50
Fig 3d-f	0.001	$[10,10^7]$	5000	50	$[8.3\times10^{-17}, 8.3\times10^{-13}]$	50

Sup Table 2. Parameter values used for the model of two consecutive reversible binding interactions.

	k_{dRS} (s^{-1})	$k_{aR,S}$ ($M^{-1}s^{-1}$)	k_{dRSD} (s^{-1})	$k_{aRS,D}$ ($M^{-1}s^{-1}$)	N_{Smax}	N_{RT}	Volume (L)	n
Fig 4, Sup Fig 3 and 4	0.001	$[10^2,10^{10}]$	0.001	$[10^2,10^{10}]$	10^4	100	1.66×10^{-13}	50

Sup Table 3. Parameter values used for the gene expression model.

	k_d (s^{-1})	k_a ($M^{-1}s^{-1}$)	k_1 (s^{-1})	k_2 (s^{-1})	d_1 (s^{-1})	d_2 (s^{-1})	N_{TFmax}	Volume (L)	n
Fig 5, Sup Fig 5 and 6	0.001	$[10^4,10^{10}]$	0.01	0.011	0.005	0.00022	10,000	1.66×10^{-14}	50
Sup Fig 7	0.001	$[10^4,10^{10}]$	0.01	0.011	0.005	0.000022	10,000	1.66×10^{-14}	50

Sup Table 4. Parameter values used for the complete signaling pathway model.

	k_{dRS} (s^{-1})	$k_{aR,S}$ ($M^{-1}s^{-1}$)	k_{dRSD} (s^{-1})	$k_{aRS,D}$ ($M^{-1}s^{-1}$)	k_1 (s^{-1})	k_2 (s^{-1})	d_1 (s^{-1})	d_2 (s^{-1})	N_{Smax}	N_{RT}	Volume (L)	n
Sup Fig 8	0.001	$[10^2,10^{10}]$	0.001	$[10^4,10^{12}]$	0.01	0.011	0.005	0.00022	5000	50	8.3×10^{-12}	25

Sup Table 5. Parameter values used for the extended gene expression model.

	k_d (s^{-1})	k_a($M^{-1}s^{-1}$)	k_{dTPD} (s^{-1})	$k_{aTD,P}$ ($M^{-1}s^{-1}$)	k_1 (s^{-1})	k_2 (s^{-1})	d_1 (s^{-1})	d_2 (s^{-1})	N_{Tmax}	N_P	Volume (L)	n
Sup Fig 9	0.001	10^6	0.001	0.0001	0.01	0.011	0.005	0.00022	100	NA	1.66×10^{-12}	25

Supplementary References

1. Sever R, Glass CK. Signaling by Nuclear Receptors. Cold Spring Harbor

Perspectives in Biology. 2013 Mar 1;5(3):a016709–a016709.
2. Lefebvre P, Benomar Y, Staels B. Retinoid X receptors: common heterodimerization partners with distinct functions. Trends in Endocrinology & Metabolism. 2010 Nov;21(11):676–83.
